# Tirzepatide and metabolic dysfunction-associated steatotic liver disease (MASLD) in obesity: a real-world multicenter study

**DOI:** 10.3389/fendo.2026.1855886

**Published:** 2026-07-06

**Authors:** Martina Galasso, Ludovica Verde, Renato Patrone, Giovanni Ragozzino, Giuseppe Annunziata, Annamaria Colao, Luigi Barrea, Giovanna Muscogiuri

**Affiliations:** 1 Centro Italiano per la cura e il Benessere del Paziente con Obesità (C.I.B.O), Unità di Endocrinologia, Diabetologia e Andrologia, Dipartimento di Medicina Clinica e Chirurgia, Università degli Studi di Napoli Federico II, Naples, Italy; 2 Department for the Promotion of Human Sciences and Quality of Life, San Raffaele Roma Open University, Rome, Italy; 3 Division of Endocrinology, Department of Medicine, The University of Arizona College of Medicine, Tucson, AZ, United States; 4 Unit of Abdominal Oncology, Division of Hepatobiliary Surgical Oncology, Istituto Nazionale Tumori,Fondazione G. Pascale, Istituto di Ricovero e Cura a Carattere Scientifico (IRCCS), Naples, Italy; 5 Ambulatorio di Endocrinologia, Diabetologia e Nutrizione Clinica, Dipartimento di Scienze e Tecnologie Ambientali, Biologiche e Farmaceutiche (DiSTABiF), Università degli studi della Campania, Caserta, Italy; 6 Dipartimento di Medicina Clinica e Chirurgia, Unità di Endocrinologia, Diabetologia ed Andrologia,Università degli Studi di Napoli Federico II, Naples, Italy; 7 Cattedra Unesco “Educazione Alla Salute E Allo Sviluppo Sostenibile”, Università degli Studi di Napoli Federico II, Naples, Italy; 8 Dipartimento di Psicologia e Scienze della Salute, Università Telematica Pegaso, Via Porzio, Naples, Italy

**Keywords:** fatty liver index (FLI), Inflammation, metabolic dysfunction–associated steatotic liver disease (MASLD), obesity, tirzepatide

## Abstract

**Introduction:**

Metabolic dysfunction-associated steatotic liver disease (MASLD) is the most common chronic liver disease worldwide and is strongly associated with obesity, insulin resistance, and systemic inflammation. Tirzepatide, a dual GIP and GLP-1 receptor agonist, has shown significant effects on weight loss and metabolic control. However, real-world evidence on hepatic steatosis remains limited. The aim of this study was to evaluate the effect of tirzepatide treatment in patients with obesity and MASLD and to identify the clinical predictors of its hepatic effect.

**Methods:**

In this retrospective study, 23 adults with overweight (BMI ≥27.0 kg/m²) with at least one weight-related comorbidity and patients with obesity (BMI ≥30.0 kg/m²) and MASLD (FLI > 44) were included. Participants received tirzepatide (2.5–5 mg weekly) for 3 months. Anthropometric, metabolic (glucose, insulin, HOMA-IR, HbA1c), lipid (cholesterol, HDL, LDL, triglycerides), inflammatory (hs-CRP), and liver enzyme (AST, ALT, GGT) parameters, together with FLI, were assessed at baseline and follow-up. Correlations of ΔFLI with clinical variables and multiple regression analyses were performed.

**Results:**

After three months of treatment, significant reductions were observed in body weight, BMI, and waist circumference (all p < 0.001). Significant improvements were also found in fasting glucose (p = 0.001), HbA1c, insulin, and HOMA-IR (all p < 0.001). Inflammatory status improved, with a significant reduction in hs-CRP (p < 0.001). Liver enzymes (AST, ALT, GGT) and lipid parameters significantly decreased (all p < 0.001) except triglycerides (p = 0.001). FLI significantly decreased from baseline to 3 months (p < 0.001). Correlation analyses showed that ΔFLI was positively associated with Δweight (p < 0.001), ΔBMI (p < 0.001), ΔWC (p = 0.002), Δhs-CRP (p = 0.007), ΔHbA1c (p = 0.045), ΔAST (p = 0.026), and ΔHDL cholesterol (p = 0.023). Multiple regression analysis identified Δhs-CRP as the only independent predictor of ΔFLI (p = 0.007), explaining 29.8% of the variance.

**Conclusions:**

In a real-world setting, tirzepatide was associated with significant improvements in MASLD along with metabolic parameters and inflammation in obesity. Improvement in MASLD was independently associated with reduced low-grade inflammation. These findings support tirzepatide as a potential therapeutic option for MASLD management in the context of obesity.

## 1 Introduction

Metabolic dysfunction-associated steatotic liver disease (MASLD) has emerged as the prevalent chronic liver condition worldwide, closely paralleling the global rise in obesity, insulin resistance, and metabolic syndrome (1).

MASLD is characterized by excessive hepatic fat accumulation in the presence of metabolic dysfunction and is increasingly recognized as a multisystem disease associated with increased cardiovascular risk, type 2 diabetes (T2D), and chronic inflammation (2). The underlying pathophysiology is complex and involves adipose tissue dysfunction that results in insulin resistance, lipotoxicity, oxidative stress, and low-grade chronic inflammation, all of which contribute to hepatic steatosis and disease progression (2).

In parallel with the growing burden of MASLD, there is an increasing need for effective therapeutic strategies targeting its underlying metabolic drivers.

Weight reduction and improvement of insulin resistance remain the cornerstone of MASLD management (3). In this context, incretin-based therapies have attracted considerable interest due to their ability to address multiple pathophysiological mechanisms simultaneously (4).

Tirzepatide, a novel dual agonist of glucose-dependent insulinotropic polypeptide (GIP) and glucagon-like peptide-1 (GLP-1) receptors, has demonstrated remarkable efficacy in promoting weight loss and improving glycemic control in individuals with obesity (5).

Its capacity to reduce adiposity, improve insulin resistance, and modulate inflammatory pathways suggests a potential role in mitigating liver fat accumulation and related metabolic damage (6).

However, despite these promising mechanisms, current clinical evidence on tirzepatide is mainly derived from *post hoc* analyses and secondary studies embedded within randomized registrational trials, particularly those included in the phase 3 SURPASS program. Among these, the SURPASS-3 MRI substudy provided some of the first dedicated evidence regarding the hepatic effects of tirzepatide (6). This multicenter, randomized phase 3 substudy enrolled 296 adults with T2D, overweight or obesity, and a fatty liver index ≥60, who were treated with tirzepatide (5, 10, or 15 mg once weekly) or insulin degludec for 52 weeks. Liver fat content (LFC), visceral adipose tissue (VAT), and abdominal subcutaneous adipose tissue (ASAT) were assessed using magnetic resonance imaging (MRI). Compared with insulin degludec, tirzepatide produced a significantly greater reduction in LFC, with pooled 10 mg and 15 mg doses achieving an absolute decrease of 8.1% versus 3.4% in the comparator group. Reductions in liver fat were also significantly correlated with decreases in body weight, VAT, and ASAT, suggesting that improvements in hepatic steatosis are closely linked to the profound metabolic effects of tirzepatide. Nevertheless, participants were selected on the basis of T2D and increased risk of fatty liver rather than a confirmed diagnosis of MASLD, and liver-related outcomes were exploratory rather than primary trial endpoints. Therefore, although these findings support a potential beneficial role of tirzepatide on hepatic steatosis, dedicated studies specifically designed to assess MASLD outcomes remain limited (6).

Consequently, the availability of dedicated data on hepatic endpoints remains limited. Moreover, evidence from routine clinical practice is still scarce, as real-world studies in this area have predominantly focused on other GLP-1 receptor agonists, such as semaglutide, leaving a gap in real-world data specifically addressing tirzepatide and liver-related outcomes (4).

Therefore, the aim of this study was to evaluate the effect of tirzepatide treatment in patients with obesity and MASLD and to identify the clinical predictors of its hepatic effect.

## 2 Methods

### Study design and population

This was a retrospective study including adults (≥ 18 years) with overweight (BMI ≥27.0 kg/m²) with at least one weight-related comorbidity and patients with obesity (BMI ≥30.0 kg/m²) and MASLD diagnosed by Fatty Liver Index (FLI) > 44, as previously reported (7) who were prescribed tirzepatide for weight management (2.5–5 mg weekly) for 3 months.

Patients with known chronic liver diseases of different etiology, including viral hepatitis and autoimmune liver disorders, significant alcohol consumption, active malignancy, pregnancy, or prior treatment with GLP-1 receptor agonists or GIP/GLP-1 dual agonists were excluded.

### Data collection

Collected data included demographic characteristics (age and sex), anthropometric measurements (weight, height, and waist circumference [WC]), and details regarding the duration and dosage of tirzepatide treatment.

Biochemical parameters assessed at baseline and follow-up included fasting plasma glucose, insulin, glycated hemoglobin (HbA1c), lipid profile (total cholesterol, HDL cholesterol, LDL cholesterol, and triglycerides), liver enzymes (aspartate aminotransferase [AST], alanine aminotransferase [ALT], and gamma-glutamyl transferase [GGT]), and high-sensitivity C-reactive protein (hs-CRP). The Homeostatic Model Assessment for Insulin Resistance (HOMA-IR) was calculated using the formula:

*HOMA-IR = (fasting plasma glucose [mg/dL] × fasting insulin [mIU/mL])/405.*

Data from a 3-month post-treatment with tirzepatide follow-up were also included. All data were entered by healthcare professionals at the respective clinical sites, based on medical records and patient-reported information obtained during routine outpatient visits.

### Assessment of metabolic dysfunction-associated steatotic liver disease (MASLD)

As previously reported (7) MASLD was diagnosed using FLI, a validated non-invasive score derived from BMI, WC, triglyceride levels, and GGT (8). FLI was calculated for each participant at baseline and after 3 months of treatment using the following formula:

*FLI = eL/(1 + eL) × 100, L = 0.953 × log_e_ TG + 0.139 BMI + 0.718 × log_e_ γGT + 0.053 × WC - 15.745*.

In accordance with previous literature, a cut-off value of FLI > 44 was considered indicative of the presence of MASLD (7).

### Statistical analysis

Data distribution was assessed using the Kolmogorov-Smirnov test. Continuous variables are reported as mean ± standard deviation (SD). Variables that were not normally distributed were log-transformed prior to analysis. Comparisons between baseline and three-month follow-up values were performed using paired Student’s t-tests for continuous variables. Correlations between FLI and anthropometric, metabolic, and inflammatory parameters at baseline and follow-up, as well as between changes in FLI (ΔFLI) and changes in other variables (Δweight loss, ΔBMI, ΔWC, Δinsulin, ΔHOMA-IR, Δhs-CRP, Δfasting plasma glucose, ΔHbA1c, ΔAST, ΔALT, ΔGGT, Δtriglycerides, Δtotal cholesterol, ΔHDL cholesterol and ΔLDL cholesterol), were calculated using Pearson’s correlation coefficient. To identify independent predictors of changes in FLI, a multiple linear regression analysis with a stepwise method was performed, including only variables that were significant in univariate analyses (p < 0.05). A two-tailed p-value <0.05 was considered statistically significant. All analyses were conducted using SPSS Statistics version 22 (IBM Corp., Armonk, NY, USA).

## 3 Results

A total of 23 participants were included in the study. Furthermore, data from all patients who completed the 3-month follow-up were included. **Table 1** summarizes the anthropometric, metabolic, inflammatory, and hepatic parameters at baseline and after 3 months of tirzepatide treatment.

**Table 1:** Anthropometric, metabolic, inflammatory, and hepatic parameters at baseline and after 3 months of tirzepatide treatment.

Parameters	Baseline (n=23)	After 3 months of tirzepatide (n=23)	Δ%	P-value
Weight (kg)	97.7 ± 10.9	85.7 ± 9.5	-12.3%	**<0.001**
BMI (kg/m²)	34.7 ± 2.7	30.4 ± 2.4	-14.0%	**<0.001**
WC (cm)	111.4 ± 5.6	101.0 ± 5.7	-9.3%	**<0.001**
Fasting plasma glucose (mg/dL)	93.6 ± 15.3	82.7 ± 7.1	-11.6%	**0.001**
Insulin (µU/mL)	34.6 ± 17.2	18.7 ± 6.4	-46.0%	**<0.001**
HbA1c (%)	5.9 ± 0.3	5.3 ± 0.2	-10.6%	**<0.001**
HOMA-IR	7.7 ± 3.2	3.8 ± 1.2	-51.0%	**<0.001**
hs-CRP (mg/L)	2.9 ± 1.4	1.6 ± 0.8	-44.0%	**<0.001**
AST (U/L)	46.3 ± 18.5	29.8 ± 10.1	-35.6%	**<0.001**
ALT (U/L)	65.1 ± 22.6	37.3 ± 11.0	-42.7%	**<0.001**
GGT (U/L)	75.0 ± 24.0	46.7 ± 15.8	-37.7%	**<0.001**
Total cholesterol (mg/dL)	193.9 ± 34.4	164.0 ± 18.4	-15.4%	**<0.001**
HDL cholesterol (mg/dL)	55.3 ± 9.7	56.0 ± 7.6	+1.2%	0.659
LDL cholesterol (mg/dL)	102.4 ± 24.8	80.7 ± 15.2	-21.2%	**<0.001**
Triglycerides (mg/dL)	181.3 ± 50.4	142.2 ± 25.3	-21.6%	**0.001**
FLI (score)	94.3 ± 3.2	77.2 ± 8.9	-18.2%	**<0.001**

A p-value in bold type denotes a significance difference (p < 0.05).

BMI, Body Mass Index; WC, Waist Circumference; hs-CRP, high-sensitivity C-Reactive Protein; HbA1c, Glycated Hemoglobin; HOMA-IR, Homeostatic Model Assessment for Insulin Resistance; AST, Aspartate Aminotransferase; ALT, Alanine Aminotransferase; GGT, Gamma-Glutamyl Transferase; HDL Cholesterol, High-Density Lipoprotein Cholesterol; LDL Cholesterol, Low-Density Lipoprotein Cholesterol; FLI, Fatty Liver Index.

After treatment with tirzepatide, a significant reduction was observed in all anthropometric parameters. In particular, body weight decreased from 97.7 ± 10.9 kg at baseline to 85.7 ± 9.5 kg after 3 months of treatment, corresponding to a 12.3% reduction (p < 0.001). Similarly, BMI decreased from 34.7 ± 2.7 kg/m² to 30.4 ± 2.4 kg/m² (−14.0%, p < 0.001), while WC declined from 111.4 ± 5.6 cm to 101.0 ± 5.7 cm (−9.3%, p < 0.001).

Significant improvements were observed in glucose metabolism parameters. Fasting plasma glucose decreased from 93.6 ± 15.3 mg/dL at baseline to 82.7 ± 7.1 mg/dL after treatment (−11.6%, p = 0.001), while HbA1c decreased from 5.9 ± 0.3% to 5.3 ± 0.2% (−10.6%, p < 0.001). Consistently, insulin levels were reduced from 34.6 ± 17.2 µU/mL to 18.7 ± 6.3 µU/mL (−46.0%, p < 0.001), and HOMA-IR was significantly reduced from 7.7 ± 3.2 to 3.8 ± 1.2 (−51.0%, p < 0.001).

A significant reduction in inflammatory status was also observed, with hs-CRP levels decreasing from 2.9 ± 1.4 mg/L to 1.6 ± 0.8 mg/L, corresponding to a 44.0% reduction (p < 0.001) after 3 months.

Regarding hepatic and lipid profiles, significant improvements were noted in AST, which decreased from 46.3 ± 18.5 U/L to 29.8 ± 10.1 U/L (−35.6%, p < 0.001), ALT, which decreased from 65.1 ± 22.6 U/L to 37.3 ± 11.0 U/L (−42.7%, p < 0.001), and GGT, which decreased from 75.0 ± 24.0 U/L to 46.7 ± 15.8 U/L (−37.7%, p < 0.001). Total cholesterol was reduced from 193.8 ± 34.4 mg/dL to 164.0 ± 18.4 mg/dL (−15.4%, p < 0.001), LDL cholesterol from 102.4 ± 24.8 mg/dL to 80.7 ± 15.2 mg/dL (−21.2%, p < 0.001), and triglycerides from 181.3 ± 50.4 mg/dL to 142.2 ± 25.3 mg/dL (−21.6%, p = 0.001). In contrast, HDL cholesterol remained stable throughout the intervention (+1.2%, p = 0.659).

Notably, FLI significantly decreased from 94.30 ± 3.17 to 77.17 ± 8.93, corresponding to an 18.16% reduction after treatment (p < 0.001).

**Table 2** shows the correlations between FLI and anthropometric, inflammatory, and biochemical parameters. At baseline, FLI was positively correlated with weight (r = 0.523, p = 0.011), BMI (r = 0.515, p = 0.012), WC (r = 0.672, p < 0.001), HOMA-IR (r = 0.426, p = 0.043), and HbA1c (r = 0.772, p < 0.001). Furthermore, significant positive correlations were found with hepatic enzymes, including AST (r = 0.433, p = 0.039), ALT (r = 0.546, p = 0.007), and GGT (r = 0.442, p = 0.035), as well as with lipid parameters such as total cholesterol (r = 0.473, p = 0.023), LDL cholesterol (r = 0.419, p = 0.046), and triglycerides (r = 0.448, p = 0.032). After 3 months of tirzepatide treatment, FLI maintained significant positive correlations with weight (r = 0.452, p = 0.030), BMI (r = 0.667, p = 0.001), WC (r = 0.653, p = 0.001), fasting plasma glucose (r = 0.535, p = 0.009), and HbA1c (r = 0.431, p = 0.040).

**Table 2:** Correlations between Fatty Liver Index (FLI) and anthropometric, inflammatory, and biochemical parameters at baseline and after 3 months of tirzepatide treatment.

Parameters	Baseline (n=23)		After 3 months of tirzepatide (n=23)	
	FLI		FLI	
	r	p-value	r	p-value
Weight (kg)	0.523	**0.011**	0.452	**0.030**
BMI (kg/m²)	0.515	**0.012**	0.667	**0.001**
WC (cm)	0.672	**<0.001**	0.653	**0.001**
hs-CRP (mg/L)	0.058	0.794	0.164	0.455
Fasting plasma glucose (mg/dL)	0.002	0.993	0.535	**0.009**
Insulin (µU/mL)	0.376	0.077	-0.216	0.322
HOMA-IR	0.426	**0.043**	-0.056	0.801
HbA1c (%)	0.772	**<0.001**	0.431	**0.040**
AST (U/L)	0.433	**0.039**	0.345	0.107
ALT (U/L)	0.546	**0.007**	0.175	0.425
GGT (U/L)	0.442	**0.035**	0.314	0.145
Total cholesterol (mg/dL)	0.473	**0.023**	0.297	0.168
HDL cholesterol (mg/dL)	0.146	0.508	0.061	0.781
LDL cholesterol (mg/dL)	0.419	**0.046**	0.144	0.512
Triglycerides (mg/dL)	0.448	**0.032**	0.381	0.073

A p-value in bold type denotes a significance difference (p < 0.05).

BMI, Body Mass Index; WC, Waist Circumference; hs-CRP, high-sensitivity C-Reactive Protein; HbA1c, Glycated Hemoglobin; HOMA-IR, Homeostatic Model Assessment for Insulin Resistance; AST, Aspartate Aminotransferase; ALT, Alanine Aminotransferase; GGT, Gamma-Glutamyl Transferase; HDL Cholesterol, High-Density Lipoprotein Cholesterol; LDL Cholesterol, Low-Density Lipoprotein Cholesterol; FLI, Fatty Liver Index.

The correlations between changes in FLI (**Δ**FLI) and changes in anthropometric, inflammatory, and biochemical parameters are reported in **Table 3**. **Δ**FLI showed significant positive correlations with **Δ**weight (r = 0.702, p < 0.001), **Δ**BMI (r = 0.674, p < 0.001), **Δ**WC (r = 0.619, p = 0.002), **Δ**hs-CRP (r = 0.546, p = 0.007), **Δ**HbA1c (r = 0.422, p = 0.045), **Δ**AST (r = 0.464, p = 0.026), and **Δ**HDL cholesterol (r = 0.471, p = 0.023).

**Table 3:** Correlation between changes in Fatty Liver Index (FLI) and changes in anthropometric, inflammatory, and biochemical parameters after 3 months of tirzepatide treatment.

Parameters	ΔFLI	
	r	*p-value
ΔWeight loss (kg)	0.702	**<0.001**
ΔBMI (kg/m²)	0.674	**<0.001**
ΔWC (cm)	0.619	**0.002**
Δhs-CRP (mg/L)	0.546	**0.007**
ΔFasting glucose (mg/dL)	-0.066	0.766
ΔInsulin (µU/mL)	0.310	0.150
ΔHOMA-IR	0.252	0.246
ΔHbA1c (%)	0.422	**0.045**
ΔAST (U/L)	0.464	**0.026**
ΔALT (U/L)	0.274	0.206
ΔGGT (U/L)	0.370	0.083
ΔTotal cholesterol (mg/dL)	0.284	0.189
ΔLDL cholesterol (mg/dL)	0.070	0.751
ΔHDL cholesterol (mg/dL)	0.471	**0.023**
ΔTriglycerides (mg/dL)	0.323	0.133

A p-value in bold type denotes a significance difference (p < 0.05).

BMI, Body Mass Index; WC, Waist Circumference; hs-CRP, high-sensitivity C-Reactive Protein; HbA1c, Glycated Hemoglobin; HOMA-IR, Homeostatic Model Assessment for Insulin Resistance; AST, Aspartate Aminotransferase; ALT, Alanine Aminotransferase; GGT, Gamma-Glutamyl Transferase; HDL Cholesterol, High-Density Lipoprotein Cholesterol; LDL Cholesterol, Low-Density Lipoprotein Cholesterol; FLI, Fatty Liver Index.

**Figure 1:**
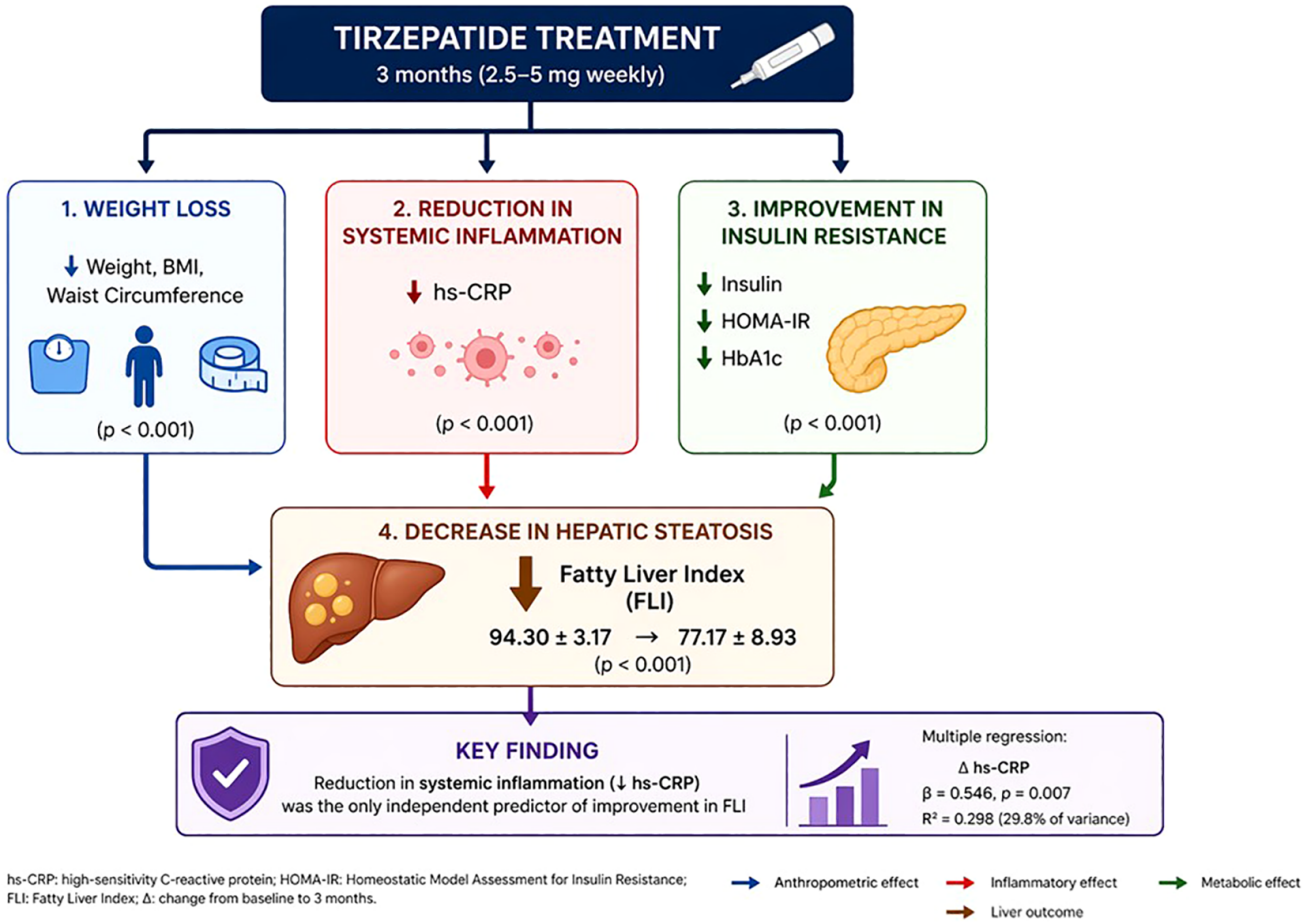
Mechanistic link between weight loss, systemic inflammation, insulin resistance, and hepatic steatosis reduction during tirzepatide treatment. Schematic representation of the interrelated mechanisms underlying the reduction in hepatic steatosis after 3 months of tirzepatide treatment in individuals with overweight with at least one weight-related comorbidity, obesity and MASLD. Tirzepatide induces significant weight loss (reduction in body weight, BMI, and waist circumference), decreases systemic inflammation (as reflected by reduced hs-CRP levels), and improves insulin resistance (lower insulin levels, HOMA-IR, and HbA1c). These interconnected metabolic and inflammatory changes contribute to the observed decrease in hepatic fat accumulation, assessed by the Fatty Liver Index (FLI). Notably, reduction in systemic inflammation (Δhs-CRP) emerged as the only independent predictor of FLI improvement in multivariable analysis, highlighting a potential central mechanistic role.

The correlations among changes in body weight, inflammatory status, insulin resistance, and FLI are summarized in **Figure 1**.

To evaluate the relative predictive power of changes in clinical parameters associated with ΔFLI, a multiple regression analysis was performed (**Table 4**). In the final model, Δhs-CRP emerged as the only significant independent predictor of ΔFLI (beta = 0.546, p = 0.007), explaining 29.8% of the variance (R^2^ = 0.298). Other variables, including ΔHOMA-IR, Δtotal cholesterol, ΔAST, and ΔALT, were excluded from the model.

**Table 4:** Multiple regression analysis model (stepwise method) with ΔFLI as the dependent variable to estimate the predictive value of changes in clinical parameters.

Parameters	Multiple Regression analysis			
	R^2^	β	t	P-value
**Δhs-CRP**	0.298	0.546	2.989	**0.007**
Variables excluded: ΔHOMA-IR, Δtotal cholesterol, ΔAST, ΔALT				
Dependent variable: ΔFLI				

A p-value in bold type denotes a significant difference (p < 0.05).

hs-CRP, high-sensitivity C-reactive protein; HOMA-IR, homeostatic model assessment of insulin resistance; AST, Aspartate Aminotransferase; ALT, Alanine Aminotransferase.

## 4 Discussion

In the present study, 3 months of tirzepatide treatment in patients with obesity and MASLD was associated with a significant reduction in FLI, suggesting a beneficial effect on MASLD. These findings directly address the primary aim of the study and support the efficacy of tirzepatide in improving MASLD-related parameters.

Evidence from randomized clinical trials further supports the hepatic efficacy of tirzepatide (6, 9, 10). In the SURPASS-3 MRI substudy, tirzepatide significantly reduced liver fat content assessed by MRI-PDFF compared with insulin degludec in patients with T2D, with reductions closely associated with decreases in body weight and visceral adiposity (6). More recently, the phase 2 SYNERGY-NASH trial demonstrated that tirzepatide significantly increased the proportion of patients achieving MASH resolution without worsening of fibrosis compared with placebo in individuals with biopsy-proven MASH and stage F2–F3 fibrosis (9). In addition, a substantial proportion of participants achieved fibrosis improvement without worsening of steatohepatitis, supporting a potential disease-modifying effect of tirzepatide in progressive fatty liver disease (9). Complementary participant-level *post hoc* analyses from the same trial further showed that histological responses were strongly associated with metabolic improvements, including greater weight loss, improved glycemic control, and normalization of liver fat, which acted as a key mediator of both MASH resolution and fibrosis improvement (10).

Real-world evidence further corroborates these findings. Sawamura et al. conducted a retrospective analysis of 40 patients with T2D and reported that switching from dulaglutide to tirzepatide was associated with significant improvements in liver enzymes, body weight, and metabolic parameters, although fibrosis scores remained largely unchanged (11). Similarly, Okuma demonstrated that switching from a GLP-1 receptor agonist to tirzepatide in 54 patients with MASLD and T2D resulted in significant reductions in body weight, FLI, and inflammatory markers, and suggesting that baseline age may predict improvements in FLI (12). Collectively, these data reinforce the role of tirzepatide in improving MASLD across both clinical trial and real-world settings.

In our cohort, changes in FLI were significantly associated with reductions in anthropometric, glycemic, and inflammatory parameters. However, in multivariable analysis, the reduction in hs-CRP emerged as the only independent predictor of FLI improvement, suggesting that systemic inflammation may play a central role in mediating hepatic benefits. Importantly, increasing evidence indicates that tirzepatide reduces inflammatory burden, as reflected by decreases in circulating inflammatory markers such as hs-CRP across clinical studies (13). These findings support the hypothesis that attenuation of low-grade systemic inflammation may represent a key pathway linking tirzepatide therapy to improvement in MASLD, beyond metabolic effects alone (14).

As expected, tirzepatide treatment resulted in substantial reductions in body weight, BMI, and WC. These findings are consistent with the well-established weight-lowering effects of tirzepatide demonstrated in randomized clinical trials and real-world studies (15, 16). Weight loss is a cornerstone in MASLD management, as excess adiposity, particularly visceral fat, is strongly associated with increased hepatic free fatty acid flux, lipotoxicity, and steatosis (17). Accordingly, the magnitude of weight reduction observed in our cohort is clinically relevant and likely contributed to the observed improvement in MASLD.

Alongside weight loss, we observed significant improvements in metabolic parameters, including fasting plasma glucose, insulin levels, HbA1c, and HOMA-IR, reflecting improved insulin resistance. Insulin resistance is a central driver of MASLD pathogenesis, promoting hepatic *de novo* lipogenesis and impairing lipid oxidation (18). Therefore, the metabolic improvements observed in our cohort likely contributed to the reduction in hepatic fat accumulation.

Finally, tirzepatide treatment resulted in significant improvements in lipid profile and liver enzymes, further supporting its favorable metabolic impact. The rapidity of these changes, observed within 3 months, suggests an early coordinated metabolic and hepatic response.

Taken together, these mechanisms—weight loss, improved insulin resistance, and reduced systemic inflammation—appear tightly interconnected and likely act synergistically (**Figure 1**). Beyond systemic metabolic effects, incretin-based therapies may also exert indirect hepatic benefits through modulation of adipose tissue function and the gut–liver axis, despite limited direct hepatic GLP-1 receptor expression (19). A positive correlation was also observed between ΔFLI and ΔHDL. Although HDL cholesterol remained overall unchanged, this finding may be explained by the biphasic HDL response described during weight-loss interventions, with an initial reduction during active weight loss followed by a later increase during weight stabilization (20). Therefore, the observed correlation likely reflects inter-individual variability in HDL remodeling at this early time point rather than a direct effect of hepatic fat reduction on HDL levels (20). Consistently, longer follow-up studies such as the SURMOUNT trials have reported more evident HDL improvements, likely because HDL levels had sufficient time to stabilize and increase (21).

The present study has several limitations. First, the retrospective design and small sample size limit generalizability. Second, the absence of a control group precludes causal inference. Third, the short follow-up does not allow evaluation of long-term hepatic outcomes. Finally, the use of FLI as a surrogate marker represents an important limitation. While FLI is a validated non-invasive index of hepatic steatosis, it does not provide information on fibrosis or histological severity. Moreover, FLI incorporates anthropometric variables such as BMI and WC, which are directly modified by tirzepatide treatment. This may lead to an overestimation of hepatic improvement, as reductions in FLI may partly reflect weight loss rather than direct changes in liver fat content. Therefore, caution is warranted in interpreting FLI changes as an isolated indicator of MASLD improvement.

## 5 Conclusion

In conclusion, this study provides novel real-world evidence that tirzepatide is associated with significant improvements in hepatic steatosis in individuals with obesity and MASLD. Beyond its well-known effects on weight loss and metabolic control, the reduction in systemic inflammation emerged as an independent predictor of hepatic improvement, highlighting a potential key mechanistic pathway. However, given the observational design, these findings should be interpreted as associative rather than causal. Further studies are warranted to confirm these results and to assess long-term hepatic outcomes.

## Data Availability

The raw data supporting the conclusions of this article will be made available by the authors, without undue reservation.

## References

[1] Miller DM McCauley KF Dunham-Snary KJ . Metabolic dysfunction-associated steatotic liver disease (MASLD): Mechanisms, clinical implications and therapeutic advances. Endocrinol Diabetes Metab. (2025) 8:e70132. doi: 10.1002/edm2.70132 41255342 PMC12627968

[2] Gallo G Nalli G Baratta F Desideri G Savoia C . Metabolic dysfunction-associated steatotic liver disease: A silent driver of cardiovascular risk and a new target for intervention. Int J Mol Sci. (2025) 26. doi: 10.3390/ijms26168081 40869400 PMC12386202

[3] Sheikh MY Younus MF Shergill A Hasan MN . Diet and lifestyle interventions in metabolic dysfunction-associated fatty liver disease: A comprehensive review. Int J Mol Sci. (2025) 26. doi: 10.3390/ijms26199625 41096891 PMC12524441

[4] Suki M Amer J Milgrom Y Massarwa M Hazou W Tiram Y . Semaglutide in MASLD patients: Improved survival and liver outcomes. Pharm (Basel). (2025) 18. doi: 10.3390/ph18071075 40732362 PMC12300756

[5] Sinha R Papamargaritis D Sargeant JA Davies MJ . Efficacy and safety of tirzepatide in type 2 diabetes and obesity management. J Obes Metab Syndr. (2023) 32:25–45. doi: 10.7570/jomes22067 36750526 PMC10088547

[6] Gastaldelli A Cusi K Fernandez Lando L Bray R Brouwers B Rodriguez A . Effect of tirzepatide versus insulin degludec on liver fat content and abdominal adipose tissue in people with type 2 diabetes (SURPASS-3 MRI): A substudy of the randomised, open-label, parallel-group, phase 3 SURPASS-3 trial. Lancet Diabetes Endocrinol. (2022) 10:393–406. doi: 10.1016/s2213-8587(22)00070-5 35468325

[7] Crudele L De Matteis C Novielli F Di Buduo E Petruzzelli S De Giorgi A . Fatty liver index (FLI) is the best score to predict MASLD with 50% lower cut-off value in women than in men. Biol Sex Differ. (2024) 15:43. doi: 10.1186/s13293-024-00617-z 38760802 PMC11100212

[8] Bedogni G Bellentani S Miglioli L Masutti F Passalacqua M Castiglione A . The fatty liver index: A simple and accurate predictor of hepatic steatosis in the general population. BMC Gastroenterol. (2006) 6:33. doi: 10.1186/1471-230x-6-33 17081293 PMC1636651

[9] Loomba R Hartman ML Lawitz EJ Vuppalanchi R Boursier J Bugianesi E . Tirzepatide for metabolic dysfunction-associated steatohepatitis with liver fibrosis. N Engl J Med. (2024) 391:299–310. doi: 10.1056/nejmoa2401943 38856224

[10] Caussy C Cusi K Rosenstock J Bugianesi E Thomas MK Tang Y . Relationship between metabolic and histological responses in people with metabolic dysfunction- associated steatohepatitis with and without type 2 diabetes: Participant-level exploratory analysis of the SYNERGY-NASH trial with tirzepatide. Diabetes Care. (2025) 48:2074–83. doi: 10.2337/dc25-1306 41066427 PMC12635880

[11] Sawamura T Mizoguchi R Ohmori A Kometani M Yoneda T Karashima S . Effects of the switch from dulaglutide to tirzepatide on glycemic control, body weight, and fatty liver: A retrospective study. J Diabetes Metab Disord. (2024) 23:2105–13. doi: 10.1007/s40200-024-01472-w 39610482 PMC11599550

[12] Okuma H . Effects of tirzepatide on patients with type 2 diabetes and metabolic dysfunction-associated steatotic liver disease: A retrospective cohort study. Cureus. (2025) 17:e83712. doi: 10.7759/cureus.83712 40486301 PMC12145500

[13] Masson W Lobo M Nogueira JP Barbagelata L Touzas P Frias JP . Anti-inflammatory effects of tirzepatide: A systematic review and meta-analysis. Rev Endocr Metab Disord. (2026) 27:5–15. doi: 10.1007/s11154-025-09991-4 41032183

[14] Boldys A Buldak L Maliglowka M Surma S Okopien B . Potential therapeutic strategies in the treatment of metabolic-associated fatty liver disease. Med (Kaunas). (2023) 59. doi: 10.3390/medicina59101789 PMC10608225 37893507

[15] Angelopoulos N Androulakis I Rizoulis A Boniakos A Fousteris E Mentzelopoulou V . A real-world study of tirzepatide for weight loss in adults without diabetes mellitus. Int J Obes (Lond). (2026) 50:684–8. doi: 10.1038/s41366-025-01986-0 41354867

[16] Hankosky ER Desai K Chinthammit C Grabner M Stockbower G He X . Real-world use and effectiveness of tirzepatide among people without evidence of type 2 diabetes in the United States. Diabetes Metab. (2025) 51:101636. doi: 10.1016/j.diabet.2025.101636 40057019

[17] Lazo M Solga SF Horska A Bonekamp S Diehl AM Brancati FL . Effect of a 12-month intensive lifestyle intervention on hepatic steatosis in adults with type 2 diabetes. Diabetes Care. (2010) 33:2156–63. doi: 10.2337/dc10-0856 20664019 PMC2945152

[18] Truong XT Lee DH . Hepatic insulin resistance and steatosis in metabolic dysfunction-associated steatotic liver disease: New insights into mechanisms and clinical implications. Diabetes Metab J. (2025) 49:964–86. doi: 10.4093/dmj.2025.0644 40935652 PMC12436041

[19] Liu QK . Mechanisms of action and therapeutic applications of GLP-1 and dual GIP/GLP-1 receptor agonists. Front Endocrinol (Lausanne). (2024) 15:1431292. doi: 10.3389/fendo.2024.1431292 39114288 PMC11304055

[20] Rolland C Broom I . The effects of very-low-calorie diets on HDL: A review. Cholesterol. (2011) 2011:306278. doi: 10.1155/2011/306278 21490771 PMC3065900

[21] Jastreboff AM Aronne LJ Ahmad NN Wharton S Connery L Alves B . Tirzepatide once weekly for the treatment of obesity. N Engl J Med. (2022) 387:205–16. doi: 10.1056/nejmoa2206038 35658024

